# Genetic Variation in Chemical Defence Affects Protection of an Herbivorous Insect Against Predation

**DOI:** 10.1111/mec.70363

**Published:** 2026-05-04

**Authors:** Johannes Körnig, Kris Ortizo, Christian Woehle, Robert Greenhalgh, Holger Schielzeth, Bruno Huettel, David G. Heckel, M. Denise Dearing, Martin Kaltenpoth, Franziska Beran

**Affiliations:** ^1^ Department of Insect Symbiosis Max Planck Institute for Chemical Ecology Jena Germany; ^2^ Population Ecology Group Friedrich Schiller University Jena Germany; ^3^ Max Planck Genome‐Centre Cologne Max Planck Institute for Plant Breeding Research Cologne Germany; ^4^ School of Biological Sciences University of Utah Salt Lake City Utah USA; ^5^ Department of Entomology Max Planck Institute for Chemical Ecology Jena Germany; ^6^ Research Group Plant‐Biotic Interactions Leibniz Institute for Vegetable and Ornamental Crops Großbeeren Germany

**Keywords:** flea beetle, gene duplication, glucosinolate, myrosinase, *Phyllotreta*, sequestration

## Abstract

Genetic variation contributes to intraspecific differences in the chemical defence in many insect species, yet the underlying genetic mechanisms remain poorly understood. The horseradish flea beetle, *Phyllotreta armoraciae* , sequesters glucosinolates from its horseradish host plant and activates them using endogenous myrosinase enzymes. Of the three known myrosinases in *P. armoraciae* , PaMyr1 functions primarily in adults, whereas PaMyr2 and PaMyr3 are responsible for myrosinase activity in larvae. Here, we identify natural genetic variation at the myrosinase locus that gives rise to three distinct myrosinase haplotypes, only one of which retains a functional *PaMyr3* gene. This variation affected *PaMyr* gene expression and myrosinase activity in larvae but not in adults. Larvae expressing both *PaMyr2* and *PaMyr3* showed elevated myrosinase activity toward 2‐propenyl glucosinolate, the major glucosinolate in horseradish. Gene expression and biochemical analyses indicate that elevated myrosinase activity results from a subfunctionalization of PaMyr3, which confers greater catalytic efficiency rather than higher total myrosinase abundance. Importantly, *PaMyr3*‐expressing larvae were less susceptible to a model generalist predator in laboratory assays, suggesting a selective advantage under high predation pressure. Consistent with this hypothesis, the *PaMyr3*‐containing haplotype occurred at higher frequency in a natural population than in long‐term laboratory populations lacking predators. Together, our results link structural genetic variation to intraspecific differences in insect chemical defence with potential consequences for predator–prey interactions in natural populations.

## 1 Introduction

Predators exert strong selection pressure on insects, driving the evolution of sophisticated antipredator strategies (Blum 1981; Nosil and Crespi 2006; Medina et al. 2025). Among these, chemical defences are exceptionally diverse and can vary substantially both within and between populations of numerous insect species (Blum 1981; Laurent et al. 2004; Mattila et al. 2021; Ottocento et al. 2023; Fernandes Erickson 2025). Such variation is predicted to have important consequences for survival by mediating interactions with predators, and thus to influence the evolutionary trajectories of species.

Intraspecific variation in chemical defences may result from environmental factors, genetic factors, or a combination of both. Environmental factors include differences in host plant chemistry which influence sequestration of plant defence compounds by insects (Brower et al. 1984; Speed et al. 2012), but also resource availability, which may constrain the de novo biosynthesis of defence metabolites (Burdfield‐Steel et al. 2019). Genetic factors have been shown to contribute to variation in the levels of biosynthesized cyanogenic glucosides in *Heliconius erato* butterflies (Mattila et al. 2021), the composition and levels of biosynthesized cardenolides in *Oreina* leaf beetles (Eggenberger and Rowell‐Rahier 1992; Triponez et al. 2007), pyrazines in the wood tiger moth *Arctia plantaginis* (Ottocento et al. 2023) and aristolochic acids sequestered by *Battus philenor* butterfly larvae from their *Aristolochia* host plants (Dimarco et al. 2012). Although genetic variation contributes to diversity in chemical defences in many insects, its genetic basis remains unknown.

In this study, we investigate the genetic basis of variation in the chemical defence of the horseradish flea beetle ( *Phyllotreta armoraciae* ). This specialist herbivore is monophagous on horseradish ( *Armoracia rusticana* ) in nature but also accepts other *Brassica* species as food plants under laboratory conditions (Nielsen et al. 1979; Vig and Verdyck 2001). Both larvae and adults sequester glucosinolates, the characteristic sulphur‐rich secondary metabolites of Brassicaceae (Sporer et al. 2020). The horseradish host plant mainly contains 2‐propenyl glucosinolate (also referred to as sinigrin), suggesting that horseradish flea beetles predominantly sequester this glucosinolate in nature (Li and Kushad 2004; Agneta et al. 2014; Ciska et al. 2017). To sequester glucosinolates, beetles must prevent the activity of plant myrosinase enzymes in ingested plant tissue, which otherwise hydrolyse glucosinolates into toxic and deterrent isothiocyanates and other biologically active metabolites (Wittstock et al. 2016; Lv et al. 2022). Horseradish flea beetles at least partially avoid hydrolysis of ingested glucosinolates by rapidly absorbing them from the gut into the haemolymph, where they are stored as part of the beetle's own chemical defence (Sporer et al. 2021; Yang et al. 2022).

To exploit sequestered glucosinolates for defence, insects must be able to hydrolyse them using endogenous myrosinases. Such enzymes have evolved from insect β‐O‐glucosidases in at least two glucosinolate‐sequestering insect lineages, *Phyllotreta* flea beetles and specialist cabbage aphids (Jones et al. 2002; Beran et al. 2014; Sun et al. 2021; Körnig et al. 2023). In *P. armoraciae* , myrosinase activity is high in larvae and adults, and has been shown to protect larvae against a generalist predator, the larvae of the Asian lady beetle, *Harmonia axyridis* (Sporer et al. 2020). Predator larvae are rapidly deterred, likely due to glucosinolate hydrolysis by beetle myrosinases in the predator gut, resulting in high larval survival (Sporer et al. 2020).

Larvae of *P. armoraciae* develop within leaf petioles and stems and are therefore primarily exposed to aboveground predators such as *H. axyridis* when the mature larvae leave the plant for pupation in the soil. In contrast, larvae of other *Phyllotreta* species typically feed on roots and are thus exposed to soil‐dwelling natural enemies including entomopathogenic nematodes and other pathogens.

Beyond their role in activated defence, beetle myrosinases also metabolize sequestered glucosinolates in uninjured larvae (Körnig et al. 2023, 2026). Silencing of myrosinase gene expression results in increased glucosinolate accumulation in larvae, but not in adults (Körnig et al. 2023), indicating life stage‐specific regulation of glucosinolate turnover. The formation of toxic hydrolysis products, such as 2‐propenyl isothiocyanate, has been proposed to contribute to defence against pathogens. Consistent with this hypothesis, glucosinolate hydrolysis products reduced the relative abundance of *Photorhabdus laumondii*, the symbiotic bacterium of the entomopathogenic nematode *Heterorhabditis bacteriophora*, but did not affect host survival upon infection (Körnig et al. 2026). Despite these insights, the natural enemy community of *Phyllotreta* flea beetles remains poorly characterized.

Whereas cabbage aphids possess a single myrosinase, *Phyllotreta* flea beetles harbour multiple myrosinases (Beran et al. 2014; Körnig et al. 2023). The horseradish flea beetle possesses three myrosinases that differ in both their biochemical properties and their expression pattern across the beetle's life cycle (Körnig et al. 2023). One myrosinase, PaMyr1, is responsible for myrosinase activity in adults and prefers 2‐propenyl glucosinolate as a substrate. A second myrosinase, PaMyr2, is active in larvae and shows a much broader substrate specificity than PaMyr1, with highest activity toward benzyl glucosinolate and 4‐methylthiobutyl glucosinolate. The third myrosinase, PaMyr3, is also expressed in larvae but appears to contribute only marginally to the total myrosinase activity at this life stage. PaMyr2 and PaMyr3 share high sequence identity (93% aa sequence identity) consistent with a recent gene duplication event (Körnig et al. 2023). Despite this high sequence similarity to PaMyr2, PaMyr3 shows a narrow substrate specificity and, similar to PaMyr1, prefers 2‐propenyl glucosinolate as a substrate. Although both larvae and adults primarily sequester 2‐propenyl glucosinolate, larvae predominantly express a myrosinase with broad substrate specificity (PaMyr2), whereas the function of PaMyr3 remains unclear.

Here, we demonstrate that not all horseradish flea beetles encode *PaMyr3* in their genome due to structural variation at the myrosinase gene locus present in both laboratory and natural beetle populations. We characterized the phenotypic consequences of this natural genetic variation under controlled conditions and show that beetle genotype affects myrosinase gene expression and activity in horseradish flea beetle larvae. Finally, using assays with a model generalist predator, we show that variation in the number of myrosinase genes affects susceptibility to predation. We demonstrate that structural genetic variation leading to the presence or absence of defence genes can drive intraspecific variation in chemical defences in herbivorous insects and thereby influence their interaction with predators.

## 2 Materials and Methods

### 2.1 Plants and Insects

The main laboratory colony of *P. armoraciae* was established in 2012 with adults collected from feral horseradish plants around Jena in Thuringia, Germany (referred to as Jena population). Field‐collected adults from the same region were added in irregular intervals to the rearing. Adults were reared on *Brassica juncea* cv. Bau‐Sin plants or *Brassica rapa* cv. Yu‐Tsai‐Sum plants (Known‐You Seeds, Kaohsiung, Taiwan) as described earlier in Sporer et al. (2020).

In August 2020, about 35 *P. armoraciae* adults were collected from feral horseradish plants in Veilsdorf, about 70 km from Jena (WGS84, N50°24′35.388″ E10°48′51.228″), to establish an additional laboratory colony (referred to as Veilsdorf population) on *B. juncea* .

Asian ladybird beetles, *H. axyridis* , were collected in Jena in Thuringia, Germany in 2020. Adults were reared on pea aphids in mesh cages within a controlled‐environment chamber at 23°C, 60% relative humidity, and a 16‐h photoperiod. Egg clutches were transferred to Petri dishes, and hatched larvae were reared in a separate cage with pea aphids. Different pea aphid clones were provided by Dr. Grit Kunert (Max Planck Institute for Chemical Ecology, Jena, Germany) and reared on potted *Vicia faba* cv. ‘The Sutton’ plants.

### 2.2 Genome Sequencing

Genomic DNA was isolated from a single *P. armoraciae* specimen of unknown sex from the Jena population reared on *B. juncea* with the MagAttract Kit (Qiagen, Hilden, Germany) and quantified using Quantus (Promega, Madison, USA). DNA quality was assessed by capillary field inversion electrophoresis using an Agilent FEMTOpulse (Agilent Technologies, Santa Clara, USA). Ten nanograms of genomic DNA were fragmented with g‐Tubes (Covaris) by passing the DNA for 3 min at 2348 × *g* through a tube. A PacBio library was prepared from 5 ng of approximately 10 kb DNA fragments following the recommendations of the protocol ‘Preparing HiFi SMRTbell Libraries from Ultra‐Low DNA Input’ (version from November 2021). The library was sequenced on a Sequel II in a single 8 M SMRT cell using Sequel Binding Kit 2.0 and Sequel Sequencing Plate 2.0 for 30 h. Demultiplexing, trimming of adapters, and deduplication of HiFi reads was done in PacBio SMRT Link v.10 using the default settings. The raw PacBio reads data have been deposited in the NCBI Sequence Read Archive under BioProject accession number PRJNA1122784.

### 2.3 Genome Assembly

HiFi reads were processed with Cutadapt v.4.6 (Martin 2011) to remove reads containing PacBio Sequel adapters, sequences from a PacBio spike‐in library, and reads below 5 kb. The processed HiFi reads (20 Gbp) were assembled using the default settings of hifiasm v.0.19.6‐r595 (Cheng et al. 2021) into two sets of contigs that can be considered as the two haplotypes. Mitochondrial sequences were identified in both haplotype assemblies with MitoHiFi v.3.2 (Uliano‐Silva et al. 2023) using the mitochondrial genome of *Phyllotreta striolata* (NCBI reference sequence NC_045901.1) as a reference. The NCBI Foreign Contamination Screen (FCS) v.0.4.0 tools FCS‐adaptor and FCS‐GX (Astashyn et al. 2024) were used to remove adaptors and identify possible contaminants. After filtering out mitochondrial and contaminant contigs, duplicate sequences in the remaining contigs were removed with purge_dups v.1.2.5 (Guan et al. 2020), utilizing alignments produced by minimap2 v.2.26‐r1175 (Li 2018). Assembly statistics and quality were assessed using assembly‐stats v.1.0.1 (https://github.com/sanger‐pathogens/assembly‐stats) and BUSCO v.5.7.1 (Manni et al. 2021) run in ‘genome’ mode using the endopterygota_odb10 database (2124 total BUSCOs). Assembly statistics and results of the BUSCO analysis are summarized in Table S1.

### 2.4 Genomic Organization of Myrosinase Genes

**FIGURE 1:**
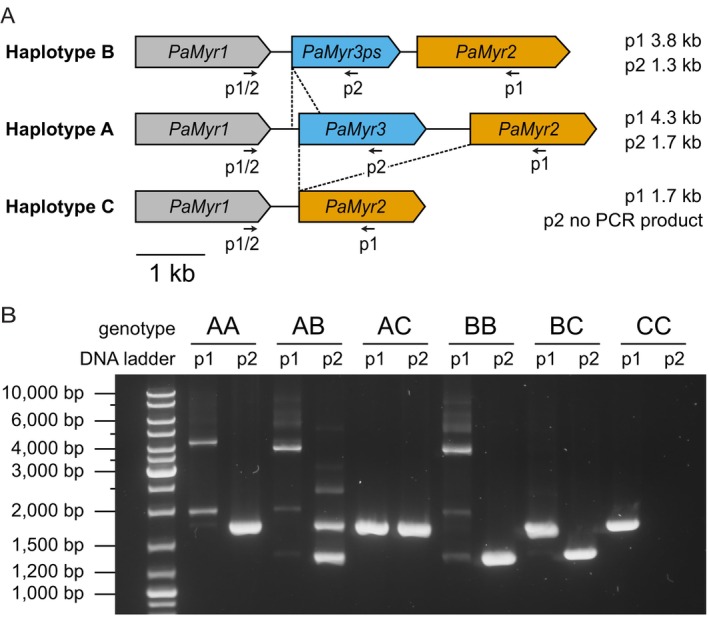
Structural variation at the myrosinase gene locus in *Phyllotreta armoraciae* . (A) Three haplotypes of the myrosinase gene locus were identified through PacBio HiFi and Sanger sequencing of the *P. armoraciae* genome. Nucleotide sequence alignments suggest that haplotypes B and C are derived from haplotype A through deletions (dashed lines). For genotyping, two separate PCR reactions were performed per DNA sample using a forward primer binding to the 3′‐end of *PaMyr1* (p1/2) in combination with reverse primers specific to *PaMyr2* (p1) or *PaMyr3* (p2). Expected PCR product sizes (shown next to the schematic) allow discrimination between homozygous and heterozygous genotypes. Genomic regions are drawn to scale. (B) Diagnostic PCR product patterns obtained with primer pairs p1 and p2 enable unambiguous identification of all six possible genotypes. In genotypes AA, AB and BB, amplification with p1 yields a large PCR product due to primer binding to *PaMyr2*, along with non‐specific PCR artifacts.

Chromosomal contigs harbouring myrosinase genes were identified in Apollo v.2.7 (Lee et al. 2013) using the BLAT nucleotide search algorithm (Kent 2002) with *PaMyr* gene sequences as queries (GenBank accession numbers OP313699–OP313701). In both assemblies, *PaMyr* genes were located in a cluster on a single contig (haplotype 1, contig 4; haplotype 2, contig 5). Manual annotation of *PaMyr* genes in Sequencher 5.4.6. revealed discrepancies in the number of encoded myrosinase genes: haplotype 1 encoded *PaMyr1*, *PaMyr2* and *PaMyr3* (later referred to as haplotype A), whereas haplotype 2 encoded only *PaMyr1* and *PaMyr2* (later referred to as haplotype C). To differentiate between these two haplotypes, we designed diagnostic primers (Table S2) that produce genotype‐specific PCR amplicon patterns (Figure 1). These primers were tested on genomic DNA extracted from individual adults using the DNeasy Blood & Tissue Kit (Qiagen). PCR amplification was performed with Phusion High‐Fidelity DNA Polymerase (Thermo Fisher Scientific, Waltham, USA) using the following program: 35 cycles of 98°C for 30 s, 57°C for 20 s and 72°C for 2 min. PCR products were visualized on 1% agarose gels. To confirm the different haplotypes by Sanger sequencing, we cloned representative amplicons into the pCR4‐TOPO vector (Thermo Fisher Scientific). Sequencing of a smaller amplicon identified a third haplotype that comprised two myrosinase genes and one myrosinase pseudogene (later referred to as haplotype B).

### 2.5 Genotype and Haplotype Frequencies in Different Laboratory and Natural Populations

To determine whether the three haplotypes discovered in the Jena population also occur in the Veilsdorf population, we extracted DNA from randomly selected adults of the Veilsdorf F1 generation (*Quick‐*DNA Tissue/Insect Microprep Kit, Zymo Research), performed PCR‐based genotyping, and cloned representative amplicons into the pCR4‐TOPO vector (Thermo Fisher Scientific) for Sanger sequencing as described above. Nucleotide sequences from different haplotypes obtained from the laboratory and Veilsdorf populations were aligned in BioEdit v.7.2.5.

To compare myrosinase genotype and haplotype frequencies between laboratory and natural beetle populations, we randomly sampled adults from the Jena populations reared on *B. juncea* (*N* = 46) and on *B. rapa* (*N* = 50), from the Veilsdorf F1 population (*N* = 56), and a natural population in Mellingen (*N* = 48, collected in August and September 2020, WGS84, N50°56′35.6892″ E11°23′3.948″). Genomic DNA was extracted from individual adults using the *Quick*‐DNA Tissue/Insect 96 Kit (Zymo Research) and PCR‐based genotyping was performed as described above. We then tested if the different populations were in Hardy–Weinberg equilibrium and compared genotype and haplotype frequencies among populations using chi‐square tests in R v.4.2.3. Pairwise comparisons were conducted with Bonferroni correction for multiple testing (Table S3).

### 2.6 Phenotypic Consequences of Structural Variation at the Myrosinase Locus in *P. armoraciae*

To analyse the phenotypic consequences of structural variation at the myrosinase gene locus in *P. armoraciae* , we established lines of all six possible genotypes (AA, AB, AC, BB, BC, CC) by crossing newly emerged, genotyped adults from the Jena population reared on *B. juncea* . For genotyping, DNA was extracted from a single hind leg and PCR‐based genotyping was performed as described above. From each line, second instar larvae and next‐generation adults were sampled for analysis of *PaMyr* transcript levels (larvae, *N =* 7–8 per line, one larva per sample; adults, 7 days old, *N* = 8 per line, one adult per sample), myrosinase activity assays (larvae, *N =* 13–15 per line, three larvae per sample; adults, 7 days old, *N* = 6 per line, two adults per sample), and glucosinolate analysis (larvae, *N* = 12–27 per line, three larvae per sample; adults, newly emerged, *N* = 15–16 per line, two to three adults per sample). Samples for enzyme activity assays and glucosinolate analysis were weighed and all samples were frozen in liquid nitrogen and stored at −80°C until extraction.

#### 2.6.1 Analysis of PaMyr Transcript Levels

*PaMyr* transcript levels were determined as described in Körnig et al. (2023). Primer efficiencies were calculated using cDNA template dilution series (Table S2). Transcript levels were normalized to the transcript level of the reference gene that demonstrated the least variability across samples, that is, ribosomal protein L18a (*RPL18a*) and eukaryotic initiation factor‐4A (*EiF4a*) for larvae, and *RPL18a* and ribosomal protein L32e (*RPL32e*) for adults.

*PaMyr* transcript levels were compared using the generalized least squares method (gls() function from the nlme library) in R v.4.2.3 (Pinheiro et al. 2021; R Core Team 2023). We accounted for sex‐specific variance differences by applying a constant variance structure using the varIdent function from the nlme library, which improved model fit. Statistical significance was determined by comparing models with and without the explanatory variable using likelihood ratio tests. Significant differences between genotypes in myrosinase transcript levels were determined by post hoc multiple comparison of estimated means using Tukey contrasts (glht() function from the multcomp library; Hothorn et al. 2020; Table S3).

#### 2.6.2 Myrosinase Activity Assays

Myrosinase activity in crude protein extracts from larvae and adults toward 2‐propenyl glucosinolate as a substrate was determined as described in Körnig et al. (2023). Myrosinase activity in larvae was compared using a generalized linear mixed model fitted with glmer() from the lme4 package (Bates et al. 2015) in R v.4.2.3. The model included extraction day as a fixed effect to account for day‐to‐day variability in measurements. The model was optimized using the BOBYQA algorithm implemented in glmer(). Significant differences among genotypes in myrosinase activity were determined by post hoc multiple comparison of estimated means using Tukey contrasts (glht () function from the multcomp library; Hothorn et al. 2020). Myrosinase activity in adults was compared using ANOVA in R v.4.2.3 (Table S3).

### 2.7 Biochemical Properties of Myrosinase Activity in AA, AC and CC Larvae

To compare myrosinase substrate specificities among AA, AC and CC larvae, we performed myrosinase activity assays using eight different glucosinolates as substrates (*N* = 3 per genotype, ten third instar larvae per replicate) as described previously in Körnig et al. (2023). 4‐Hydroxybenzyl glucosinolate was isolated from *Sinapis alba* seeds following Thies (1988) and 2‐propenyl glucosinolate was purchased from Roth (Karlsruhe, Germany). All other glucosinolates, namely 3‐butenyl, 4‐methylsulfinylbutyl, 4‐methylthiobutyl, benzyl, 2‐phenylethyl and indol‐3‐ylmethyl glucosinolate were purchased from Phytoplan (Heidelberg, Germany).

To determine the *K*_M_ values for 2‐propenyl glucosinolate, we pooled crude protein extracts from each genotype and used it in assays with substrate concentrations ranging from 0.01 to 10 mM (*N* = 3 per genotype). The estimation of *K*_M_ was performed through nonlinear regression analysis in R v.4.2.3 using drm() (Ritz et al. 2015) based on the Michaelis–Menten equation for single‐substrate reaction and the Haldane equation for single‐substrate inhibition of enzymatic reaction rate (Haldane 1930; Sonnad and Goudar 2004).

### 2.8 Glucosinolate Analysis

Glucosinolate extraction from beetle samples, conversion to desulfo‐glucosinolates, and analysis using high performance liquid chromatography coupled with diode array detection was done as described in Beran et al. (2014). Glucosinolate levels were compared using ANOVA in R v.4.2.3 (Table S3).

### 2.9 Silencing of *PaMyr3* Gene Expression

To verify that PaMyr3 is the main enzyme responsible for myrosinase activity in AA larvae, we silenced *PaMyr3* expression in AA larvae using RNA interference (RNAi). Double‐stranded RNA (dsRNA) targeting *PaMyr3* (220 bp) or a lepidopteran metalloproteinase inhibitor from *Galleria mellonella* (AY330624.1; *IMPI*, 223 bp) was synthesized using the T7 RiboMAX Express RNAi System (Promega). Putative off‐targets were identified by searching all possible 21‐mers of both RNA strands against the local *P. armoraciae* transcriptome database, allowing for a maximum of two mismatches. The putative functions of off‐target transcripts were predicted using the BLASTx algorithm by searching against the non‐redundant protein sequence database in NCBI. *PaMyr2* was identified as a putative off‐target of *dsPaMyr3* due to the high sequence similarity.

Second instar AA larvae were injected with 50 nL ultrapure water containing 80 ng dsRNA targeting *PaMyr3* or *IMPI* as a control. Injected larvae were provided with *B. juncea* leaf petioles and kept in the laboratory under ambient conditions. Six days after injection, third instar larvae were sampled and analysed for *PaMyr* transcript levels (*N* = 7 per treatment, one larva per sample), myrosinase activity assays (*N* = 8 per treatment, two larvae per sample) and glucosinolate analysis (*N* = 12 per treatment, two larvae per sample) as described above. Differences among *PaMyr* transcript levels, myrosinase activities, and glucosinolate concentrations were tested with Student's *t*‐tests in SigmaPlot v.14.0 (Table S3).

### 2.10 Predation Experiment

To test whether genetically determined variation in myrosinase activity can affect the interaction with predators, we exposed third instar *P. armoraciae* larvae of known genotypes (AA, AC and CC) to third instar larvae of the model generalist predator *H. axyridis* . One *P. armoraciae* larva and one predator larva were placed together in a 60 mm diameter Petri dish, and larval survival was recorded at 30 min intervals over a period of 6 h as described in Sporer et al. (2020). Due to the limited availability of *P. armoraciae* and *H. axyridis* larvae at the appropriate stage, predation assays were conducted on two separate days (with 3 days in between), to achieve the targeted number of replicates (*N =* 45–49 per genotype). Assays in which *H. axyridis* larvae had moulted or died were excluded from the analysis. On each assay day, control *P. armoraciae* larvae were collected for analysis of *PaMyr* transcript levels (*N =* 4 per line, one larva per sample), myrosinase activity (*N =* 6 per line, two larvae per sample) and glucosinolate analysis (*N =* 6 per line, two larvae per sample).

*PaMyr* transcript levels and glucosinolate concentrations were compared using ANOVA on ranks in SigmaPlot 14.0, and differences between genotypes were identified using the Tukey post hoc test. Myrosinase activities were compared using the generalized least squares method, accounting for genotype‐specific variance by applying a constant variance structure. Survival data were analysed using a parametric survival regression model with a lognormal hazard distribution in R 4.2.3 (Therneau 2020). The effects of larval genotype and experimental day on survival were assessed using log‐rank tests. Factor level reduction was used to determine which treatments differed from each other (Crawley 2012; Table S3).

### 2.11 Impact of Structural Variation at the Myrosinase Locus on Glucosinolate Hydrolysis Products in Uninjured Larvae

To test whether differences in myrosinase activity between AA and CC larvae affect glucosinolate metabolism in uninjured individuals, we quantified the levels of 2‐propenyl isothiocyanate and the corresponding nitrile using gas chromatography coupled with flame ionization detection (GC‐FID). Third instar larvae were collected from the AA and CC lines (*N* = 16 per line, five larvae per sample), weighed, frozen in liquid nitrogen, and stored at −80°C until extraction. Samples were extracted with 17 μL mg FW^−1^ of octane (Sigma‐Aldrich, St. Louis, USA) containing benzonitrile (1:10,000; Merck Millipore, Darmstadt, Germany) as an internal standard. Larvae were homogenized with metal beads (2 mm diameter) in a TissueLyser II (Qiagen) for 3 min at 25 Hz, during which samples gradually thawed. Homogenates were centrifuged (5 min at 16,000 × *g* at 4°C) and supernatants were transferred to plastic vials with fused inserts for GC‐FID analysis.

Samples were analysed on an Agilent 6890 GC instrument equipped with an Optima 5 capillary column (30 m × 0.25 mm i.d. × 0.25 μm film thickness; Macherey‐Nagel, Düren, Germany). The carrier gas was hydrogen and the detector temperature was 300°C. One microliter per sample was injected in splitless mode. The front inlet temperature was set to 200°C. The oven program started at 35°C (held for 4 min), increased at 12°C min^−1^ to 96°C, then at 18°C min^−1^ to 240°C (held for 6 min) and then with 60°C min^−1^ to 300°C (held for 4 min). FID response factors of 2‐propenyl isothiocyanate (1.97) and 3‐butenenitrile (1.62) relative to the internal standard benzonitrile (6.30) were calculated using the effective carbon number concept (Scanlon and Willis 1985). Authentic 2‐propenyl isothiocyanate (Sigma Aldrich Chemie GmbH, Schnelldorf, Germany) and 3‐butenenitrile (Fluka Chemie GmbH, Buchs, Switzerland) were injected as references. Concentrations of glucosinolate hydrolysis products were compared using Student's *t*‐test or the Mann–Whitney rank sum test in SigmaPlot v.14.0 (Tables S3 and S4).

### 2.12 Performance of *P. armoraciae* AA and CC Genotypes

To investigate whether genetically determined variation in myrosinase activity affects life‐history traits of *P. armoraciae* , we compared larval development time, reproductive success in terms of number of offspring over time, adult offspring weight and sex ratio, and adult sexual maturation between AA and CC lines under ad libitum food conditions (details described in Data S1). Due to space limitations, only two genotypes could be compared in this experiment.

## 3 Results

### 3.1 Genome Sequencing Reveals Structural Variation at the Myrosinase Locus

We sequenced the genome of a *P. armoraciae* beetle of unknown sex from our laboratory population using PacBio HiFi long‐read technology and analysed the organization of *PaMyr* genes in the two haplotype‐resolved genome assemblies (Data S2, Table S1). One haplotype contained all three known *PaMyr* genes, whereas the other haplotype only encoded *PaMyr1* and *PaMyr2* (Figure 1A). To confirm this structural variation at the myrosinase locus in the *P. armoraciae* genome, we designed diagnostic primers that produced haplotype‐specific PCR amplicon patterns (Figure 1B). Genotyping of individual beetles confirmed that both haplotypes are present in our laboratory population, and additionally identified a third haplotype (Figure 1, Table S2). This third haplotype carries *PaMyr1*, *PaMyr2* and a *PaMyr3*‐like pseudogene (*PaMyr3ps*) lacking the first and part of the second exon. The resulting shorter diagnostic PCR amplicon distinguishes this haplotype from the other two (Figure 1B). We refer to the three haplotypes as follows: haplotype A with three *PaMyr* genes, haplotype B with *PaMyr1*, *PaMyr2* and *PaMyr3ps* and haplotype C with only *PaMyr1* and *PaMyr2*. Nucleotide sequence alignments indicate that haplotypes B and C are derived from haplotype A through deletion events (Figure 1A). Together, our results reveal structural variation at the myrosinase locus in the *P. armoraciae* genome, with only one of the three haplotypes retaining a functional *PaMyr3* gene.

### 3.2 Structural Variation at the Myrosinase Locus Occurs in Natural Populations

To determine whether the haplotypes discovered in our laboratory population also occur in natural populations, we collected adult beetles from two locations in central Germany, approximately 70 km apart (Veilsdorf and Mellingen). All three haplotypes were detected in both field populations, and sequencing of PCR amplicons from Veilsdorf showed at least 99% nucleotide sequence identity to those of the laboratory population. These differences are predicted to result in two amino acid substitutions in PaMyr3 in the A haplotype.

**FIGURE 2:**
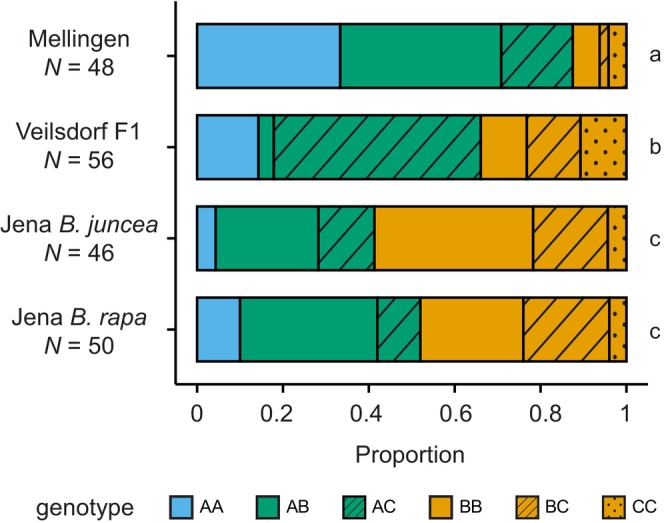
Myrosinase genotype frequencies, determined by PCR‐based genotyping of randomly sampled adult beetles, differed among natural and laboratory reared populations of *Phyllotreta armoraciae* (*p* < 0.05; results of statistical analyses are provided in Table S3). Mellingen: Adults collected from horseradish plants in Mellingen (Thuringia, Germany); Veilsdorf F1: Laboratory‐reared F1 generation derived from a natural population collected in Veilsdorf (Thuringia, Germany); Jena *Brassica juncea* and Jena *Brassica rapa* : Laboratory populations reared on *B. juncea* or *B. rapa* , respectively, and originating from the laboratory colony established in 2012 with adults collected in the Jena area (Thuringia, Germany). Colour code for *PaMyr3* copy number per genotype: Blue, two copies of *PaMyr3*; green, one copy of *PaMyr3*; yellow, no *PaMyr3* copy. Different lowercase letters indicate statistically significant differences between populations.

We then analysed haplotype and genotype frequencies in four different *P. armoraciae* populations by genotyping randomly sampled individuals from each population (*N* = 48–56 per population): two laboratory populations that had been reared separately on *B. juncea* or *B. rapa* for approximately 5 years, the first offspring generation of beetles collected from horseradish in Veilsdorf (Veilsdorf F1), and beetles collected from horseradish in Mellingen. Both genotype and haplotype frequencies varied among populations (genotype: *χ* ^2^ = 76.01, df = 15, *p* < 0.001, Figure 2; haplotype: *χ* ^2^ = 70.62, df = 6, *p* < 0.001, Figure S1). Specifically, haplotype A occurred more frequently in the Mellingen population than in either of the two laboratory populations. The Veilsdorf F1 population displayed an intermediate frequency of haplotype A relative to Mellingen and the laboratory populations (genotype AA/AB/AC vs. BB/BC/CC, *χ* ^2^ = 24.13, df = 3, *p* < 0.001). Notably, genotype frequencies deviated from Hardy–Weinberg equilibrium only in the Veilsdorf F1 population (Table S3).

### 3.3 Structural Variation at the Myrosinase Locus Influences Myrosinase Activity in Larvae

**FIGURE 3:**
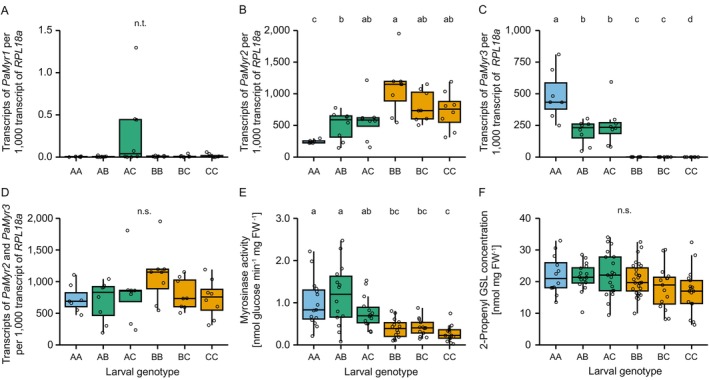
Phenotypic characterization of *Phyllotreta armoraciae* larvae with different myrosinase genotypes. *PaMyr1* transcript levels (A) in larvae were very low across all genotypes and were therefore not compared. Transcript levels of *PaMyr2* (B) and *PaMyr3* (C) differed among genotypes, whereas combined *PaMyr2* and *PaMyr3* transcript levels (D) did not differ. For statistical analysis of *PaMyr3* expression, the outlier in the AC group was excluded (but is still shown in panel C). Myrosinase activity toward 2‐propenyl glucosinolate in crude protein extracts (E) differed among genotypes, whereas levels of sequestered 2‐propenyl glucosinolate (GSL) in larvae (F) did not. Colour code for *PaMyr3* copy number per genotype: Blue, two copies of *PaMyr3*; green, one copy of *PaMyr3*; yellow, no *PaMyr3* copy. Different lowercase letters indicate statistically significant differences between genotypes (*p* < 0.05; results of statistical analyses are provided in Table S3). n.t., not tested.

To investigate the phenotypic consequences of structural variation at the myrosinase locus in *P. armoraciae* , we established beetle lines representing all six possible genotypes. Profiling of *PaMyr* transcript levels and myrosinase activity revealed genotype‐specific differences in larvae, but not in adults (Figure 3, Figure S2; statistical results are summarized in Table S3). As expected, all larval genotypes expressed *PaMyr2* (Figure 3B). In contrast, *PaMyr3* expression was detected only in AA, AB and AC genotypes (Figure 3C), consistent with the fact that only the A haplotype encodes a functional *PaMyr3* gene (Figure 1).

*PaMyr3* transcript levels were higher in AA larvae than in AB larvae but did not differ significantly between AC and either AA or AB larvae (Figure 3C). However, after excluding one outlier in the AC group, *PaMyr3* expression was significantly higher in AA larvae compared to both AB and AC larvae (Figure 3C). Notably, transcript levels of *PaMyr2* and *PaMyr3* were inversely correlated, resulting in similar combined *PaMyr2* and *PaMyr3* transcript levels across larval genotypes (Figure 3D).

Differences in the relative expression of *PaMyr2* and *PaMyr3* resulted in variation in larval myrosinase activity, which differed up to 3.7‐fold among genotypes. Myrosinase activity was higher in AA and AB larvae compared to BB, BC and CC larvae. AC larvae also showed higher activity than CC larvae (Figure 3E). Levels of sequestered glucosinolates did not differ among larval genotypes (Figure 3F).

### 3.4 PaMyr3 Contributes Substantially to Myrosinase Activity in AA Larvae

**FIGURE 4:**
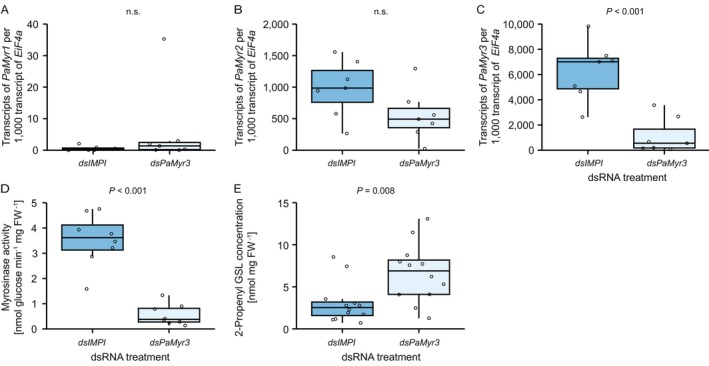
Silencing of *PaMyr3* in *Phyllotreta armoraciae* AA larvae reduces myrosinase activity and increases glucosinolate concentration. Larvae were injected with double‐stranded RNA targeting *PaMyr3* (*dsPaMyr3*) or the lepidopteran‐specific induced metalloprotease inhibitor gene (*dsIMPI*) as a control. Transcript levels of *PaMyr1* (A) and *PaMyr2* (B) did not differ between *dsIMPI*‐ and *dsPaMyr3*‐injected larvae. In contrast, ds*PaMyr3* injection reduced *PaMyr3* transcript levels (C) and myrosinase activity toward 2‐propenyl glucosinolate in crude protein extracts (D), and resulted in higher levels of sequestered 2‐propenyl glucosinolate (GSL) levels in larvae (E). Results of statistical analyses are provided in Table S3. n.s., not significant.

To determine whether PaMyr3 is responsible for the elevated myrosinase activity observed in AA larvae (Figure 3E), we silenced *PaMyr3* expression using RNAi. Transcript profiling showed that *PaMyr3* silencing did not affect *PaMyr1* transcript levels (Figure 4A) and only weakly influenced *PaMyr2* transcript levels (Figure 4B; *p* = 0.084), whereas *PaMyr3* transcript levels were reduced by 82% (Figure 4C). Silencing of *PaMyr3*, together with weak silencing of *PaMyr2*, reduced myrosinase activity by 84% and led to an increase in glucosinolate accumulation relative to control larvae (Figure 4D,E).

### 3.5 Genotype‐Dependent Variation in Myrosinase Biochemistry

To assess how genotype‐specific expression of *PaMyr2* and *PaMyr3* influences the biochemical properties of myrosinase activity, we analysed substrate specificity and Michaelis–Menten constants (*K*_M_) for 2‐propenyl glucosinolate in crude protein extracts from AA, AC and CC larvae. Protein extracts from AA larvae showed the highest activity with 2‐propenyl glucosinolate, whereas CC larvae showed the highest catalytic activity with benzyl glucosinolate and 4‐methylthiobutyl glucosinolate. AC larvae showed an intermediate substrate profile, with similar activities across all glucosinolates tested (Figure S3A–D). The *K*_M_ values for 2‐propenyl glucosinolate also differed between genotypes. Crude extracts from CC larvae had the highest apparent *K*_M_ (1.23 mM), followed by AC (0.60 mM) and AA (0.46 mM) larvae (Figure S3E–G).

### 3.6 Structural Variation at the Myrosinase Locus Affects Susceptibility to Predation

**FIGURE 5:**
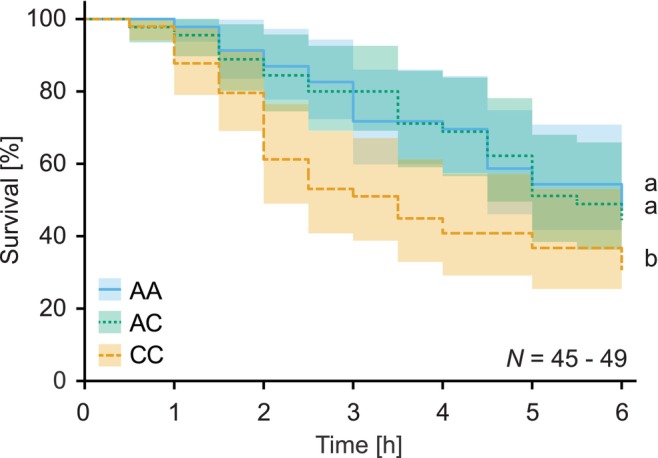
*Phyllotreta armoraciae* larvae expressing *PaMyr3* (AA and AC genotypes) survived better than CC larvae when exposed to the model generalist predator *Harmonia axyridis* (shaded area corresponds to 95% confidence interval). Different lowercase letters indicate statistically significant differences between genotypes (*p* < 0.05; results of statistical analyses are provided in Table S3).

To examine whether structural variation at the myrosinase locus influences susceptibility to predation, we exposed AA, AC and CC larvae to the model generalist predator *H. axyridis* . AA and AC larvae, which exhibit high myrosinase activity, survived better than CC larvae, which have low myrosinase activity (Log‐rank test, genotype, *χ* ^2^ = 8.65, *p* = 0.013, Figure 5, Figure S4, Table S3). This result demonstrates that structural variation at the myrosinase gene locus resulting in the presence or absence of PaMyr3 can influence the probability that *P. armoraciae* larvae survive a predator attack.

### 3.7 Structural Variation at the Myrosinase Locus Influences Glucosinolate Hydrolysis Products in Larvae

Since myrosinases are also active in uninjured larvae (Körnig et al. 2023, 2026), we compared the levels of 2‐propenyl glucosinolate hydrolysis products in AA and CC larvae using GC‐FID. Both 2‐propenyl isothiocyanate and its corresponding nitrile were detected, with the isothiocyanate accounting for 90% of the total hydrolysis products. AA larvae contained higher concentrations of 2‐propenyl isothiocyanate than CC larvae, whereas nitrile levels did not differ between genotypes (Figure S5A, Tables S3 and S4).

### 3.8 Structural Variation at the Myrosinase Locus Does Not Influence Performance Under Laboratory Conditions

To determine whether there are genotype‐specific differences in life‐history traits, we compared several parameters including the developmental time from egg until adult eclosion, the weight of newly eclosed males and females and the total number of offspring over time between AA and CC lines under ad libitum food conditions. None of the parameters analysed differed between genotypes (Figure S5B,C; Tables S3 and S5).

## 4 Discussion

Several studies have demonstrated that genetic variation contributes to intraspecific differences in insect chemical defences (Eggenberger and Rowell‐Rahier 1992; Camara 1997; Triponez et al. 2007; Dimarco et al. 2012; Mattila et al. 2021). However, the genetic mechanisms and specific genomic loci responsible for this variation remain unidentified. Here, we identify structural variation in the genome of the horseradish flea beetle, *P. armoraciae* , that underlies intraspecific variation in larval chemical defence. Structural genetic variation results in the presence or absence of *PaMyr3*, which influences larval myrosinase activity and the interaction with a generalist predator. Our findings demonstrate that structural genetic variation arising from gene duplications and deletions can generate phenotypic variation within insect populations that have the potential to alter predator–prey interactions.

The evolution of myrosinase activity in glucosinolate‐sequestering insects represents a key adaptation, enabling them to exploit host plant defence for their own benefit (Kazana et al. 2007; Beran et al. 2014; Sporer et al. 2020). Previous research identified three myrosinases in the horseradish flea beetle (Körnig et al. 2023), two of which were previously shown to be responsible for myrosinase activity in larvae and adults of a laboratory population. The function of the third myrosinase, PaMyr3, remained unclear due to its low transcript levels, suggesting only a minor contribution to the total myrosinase activity in larvae (Körnig et al. 2023). Here, we show that not all larvae can express *PaMyr3* (Figure 3C), as the gene can be absent (haplotype C) or rendered non‐functional by a 5′ truncation (haplotype B) in two of the three naturally occurring haplotypes (Figure 1A). Only haplotype A encodes a functional *PaMyr3* gene, and this haplotype was less frequent in beetles in our laboratory populations than in wild‐caught beetles (Figure 2, Figure S1). Furthermore, *PaMyr3* transcript levels were higher in homozygous AA larvae than in heterozygous AB and AC larvae (Figure 3C). The low *PaMyr3* transcript levels reported in the previous study are therefore likely explained by a low frequency of haplotype A, and in particular the AA genotype, in the laboratory population.

Gene duplications are major drivers of evolutionary innovation, enabling the neo‐ or subfunctionalization of duplicated gene copies (Ohno 1970; Zhang 2003). In addition to functional divergence, gene duplications can also increase transcript and protein abundance, which themselves may be adaptive (Puinean et al. 2010; Bass et al. 2013; Klure et al. 2025). In *P. armoraciae* , a recent gene duplication gave rise to *PaMyr2* and *PaMyr3* (Körnig et al. 2023), and larvae expressing both genes showed higher myrosinase activity with 2‐propenyl glucosinolate, the major glucosinolate beetles sequester from horseradish, than larvae expressing *PaMyr2* alone (Figure 3E). This elevated activity could stem from increased enzyme amounts due to higher total *PaMyr* transcript levels, from greater catalytic efficiency of PaMyr3, or from both mechanisms. Because *PaMyr2* expression was reduced in larvae expressing *PaMyr3* (Figure 3B), combined *PaMyr2* and *PaMyr3* transcript levels did not differ between genotypes (Figure 3D), suggesting potential dosage compensation between the two paralogs. However, PaMyr3 showed higher substrate specificity and a lower *K*_M_ value for 2‐propenyl glucosinolate compared to PaMyr2 both in vitro (Körnig et al. 2023) and in vivo (Figure S3). Our results thus indicate that elevated myrosinase activity toward 2‐propenyl glucosinolate in larvae carrying the A haplotype is primarily due to a subfunctionalization of *PaMyr3*. This functional divergence may reflect a biochemical adaptation of *P. armoraciae* to the major glucosinolate present in its horseradish host plant.

The quantity of defensive compounds an insect produces or sequesters can determine whether the individual survives a predator attack. One of the best‐known examples is the monarch butterfly, whose unpalatability to bird predators depends on the abundance and composition of cardenolides in its larval host plant (Brower et al. 1967, 1984). In aposematic species like the monarch, less‐defended individuals benefit from the presence of better defended conspecifics, as predators learn to associate warning coloration with unpalatability (Bowers 1992; Speed et al. 2012). In *P. armoraciae* , structural variation at the myrosinase locus influences the chemical defence of larvae, which are visually inconspicuous and do not advertise their unpalatability to predators. Previous predation experiments have shown that horseradish flea beetle larvae can survive attacks by *H. axyridis* larvae by rapidly deterring this generalist predator, suggesting that individual‐level selection has played an important role in the evolution of this chemical defence (Sporer et al. 2020). Here, we demonstrate that genetic variation in myrosinase activity can further influence this interaction: AA and AC larvae, which exhibit higher myrosinase activity, survived longer in our predation assay than CC larvae with lower activity. Because surviving the initial encounter with a predator is critical, the differences observed in our laboratory assays are likely to translate into higher survival under natural conditions, but further studies with ecologically relevant natural enemies are needed to understand the influence of this genetic variation on predator–prey interactions.

Sequestered glucosinolates appear to be hydrolysed not only during predator attack but also in uninjured *P. armoraciae* larvae and adults (Sporer et al. 2021; Körnig et al. 2023, 2026), a process we refer to as endogenous glucosinolate metabolism. Our findings provide additional evidence for this process: *PaMyr3*‐silenced larvae with low myrosinase activity accumulated more glucosinolates than control larvae (Figure 4E), and uninjured AA larvae with high myrosinase activity contained more isothiocyanates than CC larvae with low myrosinase activity (Figure S5A). The detection of unmetabolized isothiocyanates in larval bodies is particularly interesting, considering that these metabolites are highly reactive and therefore toxic to a wide range of organisms. In generalist lepidopteran larvae, isothiocyanate metabolism via conjugation with glutathione incurs high physiological costs that suppress larval growth (Jeschke et al. 2016). Beyond the impact on the relative abundance of *P. laumondii* bacteria in *P. armoraciae* larvae infected with entomopathogenic nematodes (Körnig et al. 2026), the function of endogenous glucosinolate metabolism in *P. armoraciae* remains unknown, as does the metabolic fate of the reactive isothiocyanates produced. Although specialized and well‐adapted herbivores such as *P. armoraciae* likely tolerate such metabolites and may even exploit them for nutritional benefits or defence against pathogens, the use of such chemical defences may still be associated with physiological costs.

Physiological costs and variable predation pressure are considered major drivers of quantitative variation in chemical defences within and among insect populations (Skelhorn and Ruxton 2008; Speed et al. 2012). Our findings suggest that beetles exhibiting higher myrosinase activity due to PaMyr3 may gain a selective advantage under high predation pressure. Consistent with this hypothesis, we found a higher proportion of beetles carrying *PaMyr3* in a natural population compared with two long‐term laboratory populations that are not exposed to predators. Moreover, the deviation from Hardy–Weinberg equilibrium observed in the Veilsdorf F1 population may reflect a sudden shift in selection pressure associated with the transition to predator‐free laboratory conditions.

We detected no difference in the performance of AA and CC genotypes under laboratory conditions. However, our experimental conditions did not allow for the detection of growth‐defence trade‐offs, as larvae may have compensated for potential physiological costs by increasing their feeding rates (Simpson and Simpson 1990; Cloutier et al. 2000). Thus, the persistence of haplotypes B and C in populations remains unexplained, and further work is needed to identify the ecological and evolutionary factors maintaining this genetic polymorphism and shaping genotype and haplotype frequencies in *P. armoraciae* populations.

In summary, our study demonstrates that structural genetic variation, resulting in the presence or absence of a functional defence gene in the beetle genome, leads to quantitative differences in larval chemical defence in the horseradish flea beetle. Predation pressure is likely an important selective force favouring highly defended genotypes; however, the persistence of less‐defended genotypes suggests that additional ecological factors contribute to the maintenance of this polymorphism. Overall, our findings highlight gene duplications and losses as major sources of variation in insect chemical defences and provide a foundation for mechanistic studies aimed at understanding how and why genetic diversity in chemical defence is maintained in nature.

## Author Contributions

J.K., R.G., D.G.H. and F.B. designed the experiments. J.K., K.O., C.W., R.G. and F.B. performed research. H.S., M.D.D., D.G.H., B.H. and M.K. contributed new reagents/analytic tools. J.K., K.O., C.W., R.G., H.S., D.G.H. and F.B. analysed data. J.K. and F.B. wrote the paper.

## Funding

This study was supported by Deutsche Forschungsgemeinschaft (501638262) and Max‐Planck‐Gesellschaft.

## Disclosure

Benefits from this research accrue from the sharing of our data and results on public databases as described above.

## Conflicts of Interest

The authors declare no conflicts of interest.

## Supporting information


**Data S1:** Methods: Comparison of life‐history traits of *Phyllotreta armoraciae* AA and CC genotypes.
**Data S2:** Results: Genome assembly of *Phyllotreta armoraciae*.
**Figure S1:** Comparison of myrosinase haplotype frequencies in natural and laboratory reared populations of *Phyllotreta armoraciae* .
**Figure S2:** Phenotypic characterization of *Phyllotreta armoraciae* adults with different myrosinase genotypes.
**Figure S3:** Myrosinase activity in crude protein extracts of larvae with different genotypes.
**Figure S4:** Variation in PaMyr transcript levels and myrosinase activity among genotypes of *Phyllotreta armoraciae* larvae used in predation assays.
**Figure S5:** Glucosinolate hydrolysis products in AA and CC larvae and life‐history traits of AA and CC lines of *Phyllotreta armoraciae* .
**Table S1:** Assembly statistics and BUSCO analyses of the two haplotype‐resolved *Phyllotreta armoraciae* genomes.
**Table S2:** Primers used in this study.
**Table S3:** Results of statistical analyses.
**Table S4:** Hydrolysis products extracted from uninjured *Phyllotreta armoraciae* larvae quantified by GC‐FID.
**Table S5:** Life‐history traits of AA and CC lines under ad libitum food conditions.

## Data Availability

The data that support the findings of this study are openly available in Edmond at https://edmond.mpg.de/, reference number https://doi.org/10.17617/3.0IXYES.
