# Supplementary material for: Genetic Variation in Chemical Defence Affects Protection of an Herbivorous Insect Against Predation
Source: Mol Ecol. 2026 May 4;35:e70363. doi: 10.1111/mec.70363 (PMC13136917; doi:10.1111/mec.70363)
Supplement: Supplementary file 1 — Data S1: Methods: Comparison of life‐history traits of Phyllotreta armoraciae AA and CC genotypes. Data S2: Results: Genome assembly of Phyllotreta armoraciae. Figure S1: Comparison of myrosinase haplotype frequencies in natural and laboratory reared populations of Phyllotreta armoraciae . Figure S2: Phenotypic characterization of Phyllotreta armoraciae adults with different myrosinase genotypes. Figure S3: Myrosinase activity in crude protein extracts of larvae with different genotypes. Figure S4: Variation in PaMyr transcript levels and myrosinase activity among genotypes of Phyllotreta armoraciae larvae used in predation assays. Figure S5: Glucosinolate hydrolysis products in AA and CC larvae and life‐history traits of AA and CC lines of Phyllotreta armoraciae . Table S1: Assembly statistics and BUSCO analyses of the two haplotype‐resolved Phyllotreta armoraciae genomes. Table S2: Primers used in this study. Table S3: Results of statistical analyses. Table S4: Hydrolysis products extracted from uninjured Phyllotreta armoraciae larvae quantified by GC‐FID. Table S5: Life‐history traits of AA and CC lines under ad libitum food conditions. [file MEC-35-e70363-s001.docx]

**Supplementary Material for:**

**Genetic variation in chemical defence affects protection of an herbivorous insect against predation**

Johannes Körnig, Kris Ortizo, Christian Woehle, Robert Greenhalgh, Holger Schielzeth, Bruno Huettel, David G. Heckel, M. Denise Dearing, Martin Kaltenpoth, Franziska Beran

Table of Contents:

| SI Methods | Page 2 |
| --- | --- |
| SI Results | Page 4 |
| Table S1: Assembly statistics and BUSCO analyses of the two haplotype-resolved *Phyllotreta armoraciae* genomes. | Page 5 |
| Table S2: Primers used in this study. | Page 6 |
| Table S3: Results of statistical analyses. | Page 7 |
| Table S4: Hydrolysis products extracted from uninjured *Phyllotreta armoraciae* larvae quantified by GC-FID. | Page 10 |
| Table S5: Life-history traits of AA and CC lines under *ad libitum* food conditions. | Page 11 |
| Figure S1: Comparison of myrosinase haplotype frequencies in natural and laboratory reared populations of *Phyllotreta armoraciae*. | Page 12 |
| Figure S2: Phenotypic characterization of *Phyllotreta armoraciae* adults  with different myrosinase genotypes. | Page 13 |
| Figure S3: Myrosinase activity in crude protein extracts of larvae with different genotypes. | Page 14 |
| Figure S4: Variation in PaMyr transcript levels and myrosinase activity among genotypes of *Phyllotreta armoraciae* larvae used in predation assays. | Page 15 |
| Figure S5: Glucosinolate hydrolysis products in AA and CC larvae and life-  history traits of AA and CC lines of *Phyllotreta armoraciae* | Page 16 |
| SI References | Page 17 |

SI Methods

**Comparison of life-history traits of *Phyllotreta armoraciae* AA and CC genotypes**

Newly eclosed adults from AA and CC lines were collected over six consecutive days. Adults were sexed and weighed on the day of collection. Mating pairs were established by placing one male and one female in a Petri dish with moistened filter paper and *Brassica juncea* leaf material (third or fourth true leaf; *N* = 28-32 per line). The Petri dishes were kept under rearing conditions and were examined daily for the presence of eggs. Mating pairs were provided with fresh leaf material every other day, and adult weight was recorded after four days of feeding. At this time point, the mean female-to-male weight ratio was 1.39 ± 0.18 (mean ± SD). Pairs with female-to-male weight ratios falling outside the 1.15 to 1.6 range were excluded from the experiment to maintain similar starting conditions between AA and CC groups. The four-day post-emergence weight of females kept for the experiment was 2.98 ± 0.31 mg (mean ± SD).

Upon initial observation of egg-laying, the mating pairs were transferred to individual insect cages (cubic, 30 cm) containing one three- to four-week-old B. juncea plant (*N* = 11-12 per line) under rearing conditions. Plants were exchanged every three to four days over 67 days. These conditions ensured no resource limitation for larval development. Plants with eggs were kept in separate cages for larval development. Four weeks later, the remaining plant material was removed, and the soil containing pupae was stored in plastic containers (9 L, Lock&Lock). The mating pair beetles were frozen in liquid nitrogen, and stored at -20°C, until we confirmed the genotype (as described above). The **larval development time** was estimated based on the time until the first adult offspring emerged and was compared between lines with the Student's *t*-test in SigmaPlot v.14.0 (Table S3, Table S5).

For the initial three weeks following the emergence of the first F1 adults, adult emergence was monitored every day. Thereafter, newly eclosed adults were collected every two to three days. For each mating pair, we recorded the number, weight, and sex of each F1 adult offspring, either until 50 days after the first adult eclosion or until 40 offspring had emerged. **Reproductive success** in terms of cumulative offspring numbers over time were compared between genotypes, with maternal four-day weight included as a random factor, using a generalized linear model (GLM). After detecting overdispersion in the Poisson GLM, we fitted a quasi-GLM to obtain adjusted standard errors (Table S3).

To estimate **population growth rates** (r) for each mating pair, we fitted individual logistic growth models to the cumulative number of eclosing offspring over time, using the fit_growthmodel() function from the **growthrates** package (Petzoldt 2022). The estimated population growth rates were tested for effects of parental genotype and maternal weight by ANCOVA. Due to parental beetles escaping during the experiment, one AA replicate and two CC replicates were excluded from the analyses. All statistical analyses were performed in R v.4.2.3 (Table S3).

**Adult offspring weight** was compared between sexes and genotypes using a linear mixed-effects model implemented in the lme4 package (Bates et al. 2015). We included the parental pair as a random intercept to account for non-independence among offspring from the same parental pair and potential unmeasured variation between pairings. Model selection was guided by Akaike’s Information Criterion. The **sex ratio** of F1 adults was compared between genotypes using a Chi-square test. Statistical analyses were performed in R v.4.2.3 (Table S3, Table S5).

To assess whether female AA and CC adults differ in maturation time, we recorded the time until the onset of egg-laying as a proxy for **sexual maturation**. Mating pairs of non-siblings, newly eclosed adult offspring were established as described above (*N* = 9 per line). Petri dishes were checked daily until egg-laying was observed. Data were compared using a Mann-Whitney rank sum test in R v.4.2.3 (Table S3, Table S5).

SI Results

**Genome assembly of *Phyllotreta armoraciae***

The genomic DNA isolated from a single *P. armoraciae* beetle was sequenced using PacBio HiFi long read sequencing technology and assembled into the two haplotypes using hifiasm. Assembly statistics and results of BUSCO analyses are summarized in Table S1. With total lengths of 115.6 Mb and 110.0 Mb, the two haplotype-resolved genome assemblies were shorter than those of *Phyllotreta cruciferae* and *Phyllotreta striolata* at 135.2 Mb and 132.3 Mb, respectively (King et al. 2023). Both assemblies have high BUSCO scores with 96.2% and 97.4% complete BUSCOs respectively, which are comparable with the BUSCO completeness of the genome assemblies of *P. cruciferae* (98.7%) and *P. striolata* (98.2%).

Table S1. Assembly statistics and BUSCO analyses of the two haplotype-resolved *Phyllotreta armoraciae* genomes.

|  | **Haplotype 1** | **Haplotype 2** |
| --- | --- | --- |
| Total length | 115.6 Mb | 110.0 Mb |
| Number of contigs | 143 | 60 |
| N50 | 6.2 Mb | 6.0 Mb |
| GC content | 34.73% | 34.85% |
| Complete BUSCOs^†^ | 96.2% | 97.4% |
| Complete single-copy BUSCOs | 95.4% | 96.3% |
| Complete duplicated BUSCOs | 0.8% | 1.1% |
| Fragmented BUSCOs | 0.4% | 0.4% |
| Missing BUSCOs | 3.4% | 2.2% |

^†^n=2124 (endopterygota lineage)

Table S2. Primers used in this study.

| **Gene** | **Primer name** | **Primer sequence 5' - 3'** | **Use** | **Primer efficiency** |
| --- | --- | --- | --- | --- |
| *PaMyr1* | PaMyrI-gDNA-3fwd | CTACCATTTCGGACTTATCGGAATA | genotyping |  |
| *PaMyr2* | Myr2_spec_rev | TGTCGTTGTTCACCAACATGGTG | genotyping |  |
| *PaMyr3* | Myr3_spec_rev | GCTACCAACGCGGAATAGTAGT | genotyping |  |
| *PaMyr3* | T7_PaMyr3_fwd | ATCCTAATACGACTCACTATAGGATTAAC  CACTACTATTCCGCGTT | Amplification of DNA templates for dsRNA synthesis; fwd |  |
| *PaMyr3* | T7_PaMyr3_rev | ATCCTAATACGACTCACTATAGGCGAGAC  CCATCTCTGCGAATATA | Amplification of DNA templates for dsRNA synthesis; rev |  |
| *IMPI* | T7-IMPI-F2 | TAATACGACTCACTATAGGGAGAGTAATG  ACAAGTGCTACTGTGAAGAT | Amplification of DNA templates for dsRNA synthesis; fwd |  |
| *IMPI* | T7-IMPI-R2 | TAATACGACTCACTATAGGGAGAGGGGAG  TCAATGCAGGAAAACT | Amplification of DNA templates for dsRNA synthesis; rev |  |
| *RPL18a* | qRPL18a-F2 | GGCCACAAGTCAAACAATTCCA | qPCR; fwd | 1.91 |
| *RPL18a* | qRPL18a-R2 | AAGTAAGTTCTGGGTTTGCGGA | qPCR; rev |  |
| *RPL32e* | qRPL32e-2F | ATACTGTGCTGAAATCGCCCAT | qPCR; fwd | 1.92 |
| *RPL32e* | qRPL32e-2R | AATCTAGCGTGTCCATTGGTGA | qPCR; rev |  |
| *EiF4a* | qPaEiF4a_F | CACGGTGACATGGAGCAAAG | qPCR; fwd | 1.95 |
| *EiF4a* | qPaEiF4a_R | ACCTCTGGCCAACAAATCGG | qPCR; rev |  |
| *PaMyr1* | qPaC2654-3-F | AACGGTTACGCTGACACGAT | qPCR; fwd | 1.97 |
| *PaMyr1* | qPaC2654-3-R | ACCGCCACCATTCCGTATTT | qPCR; rev |  |
| *PaMyr2* | Parm_C41_qPCR_2_F | ACACCAGAGCTGTGATGTCG | qPCR; fwd | 1.86 |
| *PaMyr2* | Parm_C41_qPCR_2_R | ATCTCTGACACCACTCGGGA | qPCR; rev |  |
| *PaMyr3* | q-PaMyrIIb-3-fwd | ACTATTCCGCGTTGGTAGCC | qPCR; fwd | 1.82 |
| *PaMyr3* | q-PaMyrIIb-3-rev | CAGGATGGTTCGACGTCACA | qPCR; rev |  |

Table S3. Results of statistical analyses.

| **Experiment (Figure)** | **Comparison** | **Statistical Method** | **Variance structure or Cofactor** | **Variable** | ***N*** | **Statistics** | ***P*** |
| --- | --- | --- | --- | --- | --- | --- | --- |
| Genotype and haplotype frequencies among four beetle populations (Fig. 2, Fig. S1) | genotype frequencies | Chi-square test | - | genotype * population | 46-56 | *χ*² = 76.01 | < 0.001 |
|  | haplotype frequencies | Chi-square test | - | haplotype * population | 46-56 | *χ*² = 70.62 | < 0.001 |
|  | genotype frequencies of AA/AB/AC vs BB/BC/CC beetles | Chi-square test | - | genotype * population | 46-56 | *χ*² = 24.13 | < 0.001 |
| Hardy-Weinberg statistics on populations | Mellingen | Chi-square test | - | - | 48 | χ² = 3.82 | 0.282 |
|  | Veilsdorf F1 | Chi-square test | - | - | 56 | χ² = 18.77 | < 0.001 |
|  | Jena *Brassica juncea* | Chi-square test | - | - | 46 | χ² = 2.12 | 0.614 |
|  | Jena *Brassica rapa* | Chi-square test | - | - | 50 | χ² = 0.23 | 0.968 |
| Gene expression, myrosinase activity, and glucosinolate concentration in second instar larvae (Fig. 3) | *PaMyr2* transcript levels | Generalized least squares method | varIdent (form = ~ 1 \| genotype) | genotype | 7-8 | *LR* = 52.29 | < 0.001 |
|  | *PaMyr3* transcript levels | Generalized least squares method | varIdent (form = ~ 1 \| genotype) | genotype | 7-8 | *LR* = 56.26 | < 0.001 |
|  | *PaMyr3* transcript levels without outlier | Generalized least squares method | varIdent (form = ~ 1 \| genotype) | genotype | 7-8 | *LR* = 59.77 | < 0.001 |
|  | total *PaMyr2* and *PaMyr3* transcript levels | ANOVA | - | genotype | 7-8 | *F* = 1.43 | 0.234 |
|  | myrosinase activity per mg fresh weight | Generalized linear mixed model (Gamma distribution) | Cofactor: extraction day | genotype | 13-15 | *χ*² = 50.44 | < 0.001 |
|  | 2-propenyl glucosinolate amount per mg fresh weight | ANOVA | - | genotype | 12-27 | *F* = 2.15 | 0.065 |
| Gene expression, myrosinase activity, and Glucosinolate concentration in adults (Fig. S2) | *PaMyr1* gene expression | Generalized least squares method | varIdent (form = ~ 1 \| genotype) | genotype | 8 | *LR* = 4.58 | 0.469 |
|  | myrosinase activity per mg fresh weight | ANOVA | - | genotype | 6 | *F* = 0.37 | 0.868 |
|  | 2-propenyl glucosinolate amount per mg fresh weight | ANOVA | - | genotype | 15-16 | *F* = 1.17 | 0.328 |
| RNA interference in AA larvae (Fig. 4) | *PaMyr1* transcript levels | Mann-Whitney rank sum test | - | dsRNA treatment | 7 | *U* = 17.5 | 0.383 |
|  | *PaMyr2* transcript levels | Two-tailed Student's *t*-test | - | dsRNA treatment | 7 | *t* = *1.89* | 0.084 |
|  | *PaMyr3* transcript levels | Two-tailed Student's *t*-test | - | dsRNA treatment | 7 | *t* = 4.88 | < 0.001 |
|  | myrosinase activity per mg fresh weight | Two-tailed Student's *t*-test | - | dsRNA treatment | 8 | *t* = 7.66 | < 0.001 |
|  | 2-propenyl glucosinolate amount per mg fresh weight | Two-tailed Student's *t*-test | - | dsRNA treatment | 12 | *t* = 2.92 | 0.008 |
| Predation experiment with AA, AC, and CC genotype larvae (Fig. 5, Fig. S4) | *PaMyr2* transcript levels | ANOVA on ranks | - | genotype | 4 | *H* = 9.85 | < 0.001 |
|  | *PaMyr3* transcript levels | ANOVA on ranks | - | genotype | 4 | *H* = 8.77 | < 0.001 |
|  | myrosinase activity per mg fresh weight | Generalized least squares method | varIdent (form = ~ 1 \| genotype) | genotype | 6 | *LR* = 15.77 | < 0.001 |
|  | 2-propenyl glucosinolate amount per mg fresh weight | ANOVA on ranks | - | genotype | 10 | *H* = 0.42 | 0.810 |
|  | Larval survival | Log-rank-test | - | genotype | 45-49 | *χ*² = 8.65 | 0.013 |
|  |  |  |  | experiment day |  | *χ*² = 10.07 | 0.233 |
| Glucosinolate hydrolysis products in larvae  (Fig. S5 A) | 2-propenyl isothiocyanate concentration | Two-tailed Student's *t*-test | - | genotype | 16 | *t* = 2.86 | 0.008 |
|  | 3-butenenitrile concentration | Mann-Whitney rank sum test | - | genotype |  | *U* = 126.00 | 0.950 |
|  | total glucosinolate hydrolysis product concentration | Mann-Whitney rank sum test | - | genotype |  | *U* = 62.00 | 0.013 |
| Life-history traits (Fig. S5 B and C, Table S5) | time from first egg laying to first adult offspring eclosion | Two-tailed Student's *t*-test | - | genotype | 9-11 | *t* = 0.77 | 0.453 |
|  | total reproductive success | Generalized linear model (quasi-Poisson, log link) | - | genotype × weight | 9-11 | *F* = 0.53 | 0.478 |
|  |  |  |  | genotype |  | *F* = 3.30 | 0.087 |
|  |  |  |  | weight |  | *F* = 4.61 | 0.046 |
|  | reproduction rate *r* | ANCOVA | - | genotype × weight | 9-11 | *F* = 1.29 | 0.273 |
|  |  |  |  | genotype |  | *F* = 1.22 | 0.284 |
|  |  |  |  | weight |  | *F* = 1.73 | 0.205 |
|  | F1 offspring weight | Linear mixed model | Cofactor: mating pair | sex × genotype | 269-310 | *χ*² = 1.05 | 0.304 |
|  |  |  |  | sex |  | *χ*² = 601.70 | < 0.001 |
|  |  |  |  | genotype |  | *χ*² = 1.97 | 0.160 |
|  | F1 offspring sex ratio | Chi-square test | - | sex × genotype | 269-310 | *χ*² = 0.002 | 0.964 |
|  | time from F1 eclosion to first egg laying (sexual maturation) | Mann-Whitney rank sum test | - | genotype | 9 | *U* = 39.50 | 0.963 |

Table S4. Hydrolysis products extracted from uninjured *Phyllotreta armoraciae* larvae quantified by GC-FID.

|  | |  | | **Glucosinolate hydrolysis product concentration [pmol mg FW^-1^] (mean ± SD)** | | | | | |
| --- | --- | --- | --- | --- | --- | --- | --- | --- | --- |
| **Genotype** | | ***N*** | | **3-Butenenitrile** | | **2-Propenyl isothiocyanate** | | **Total** | |
| AA | | 16 | | 6.51 ± 11.97 | | 58.13 ± 16.01 | | 64.64 ± 23.41 | |
| CC | | 16 | | 4.01 ± 5.48 | | 40.96 ± 17.47 | | 44.97 ± 18.89 | |

Table S5. Life-history traits of AA and CC lines under *ad libitum* food conditions.

| **Parameter** | **Genotype** | **Sex** | ***N*** | **mean ± SD** |
| --- | --- | --- | --- | --- |
| Number of days from first egg laying to first adult offspring eclosion | AA |  | 11 | 42.82 ± 1.17 |
|  | CC |  | 9 | 42.33 ± 1.66 |
| Total reproductive success  (total offspring number) | AA |  | 11 | 426.36 ± 72.64 |
|  | CC |  | 9 | 470.22 ± 53.30 |
| Population growth over time  (reproduction rate) | AA |  | 11 | 0.11 ± 0.00 |
|  | CC |  | 9 | 0.11 ± 0.01 |
| F1 offspring weight after eclosion | AA | female | 310 | 2.38 ± 0.34 |
|  | CC | female | 287 | 2.31 ± 0.37 |
|  | AA | male | 289 | 1.85 ± 0.27 |
|  | CC | male | 269 | 1.82 ± 0.26 |
| F1 offspring sex-ratio | AA | female | 310 |  |
|  | CC | female | 287 |  |
|  | AA | male | 289 |  |
|  | CC | male | 269 |  |
| Number of days from eclosion to first egg laying (sexual maturation) | AA |  | 9 | 5.67 ± 1.22 |
|  | CC |  | 9 | 5.67 ± 0.71 |


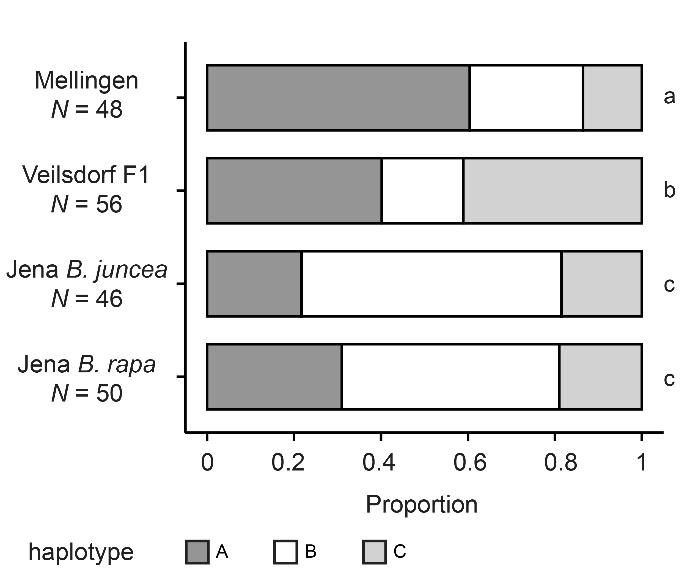


Figure S1

Myrosinase haplotype frequencies, determined by PCR-based genotyping of randomly sampled adult beetles, differed significantly among natural and laboratory reared populations of *Phyllotreta armoraciae (P* < 0.05; results of statistical analyses are provided in Table S3). Mellingen: adults collected from horseradish plants in Mellingen (Thuringia, Germany). Veilsdorf F1: laboratory-reared F1 generation derived from a natural population collected in Veilsdorf (Thuringia, Germany). Jena *B. juncea* and Jena *B. rapa*: laboratory populations reared on *Brassica juncea* or *Brassica rapa,* respectively, and originating from the laboratory colony established in 2012 with adults collected in the Jena area (Thuringia, Germany). Different lowercase letters indicate statistically significant differences between populations.


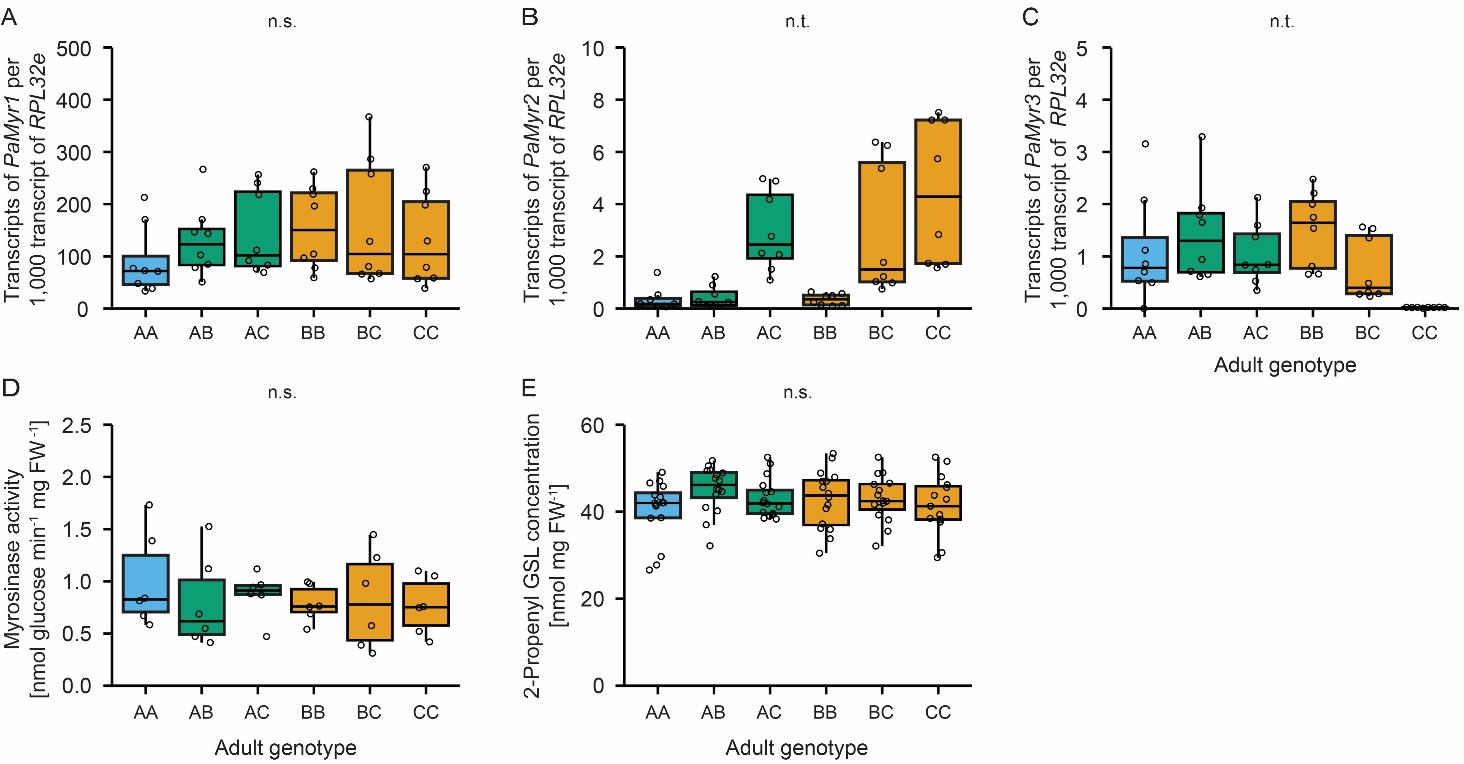


Figure S2

Phenotypic characterization of *Phyllotreta armoraciae* adults with different myrosinase genotypes. *PaMyr1* transcript levels (A) did not differ among genotypes. Transcript levels of PaMyr2 (B) and PaMyr3 (C) were low across all genotypes in adults and were therefore not statistically compared. Neither myrosinase activity toward 2-propenyl glucosinolate in crude protein extracts (D) nor the levels of sequestered 2-propenyl glucosinolate (GSL) in adults (E) differed between genotypes. Results of statistical analyses are provided in Table S3. n.s., not significant, n.t., not tested because of low expression.


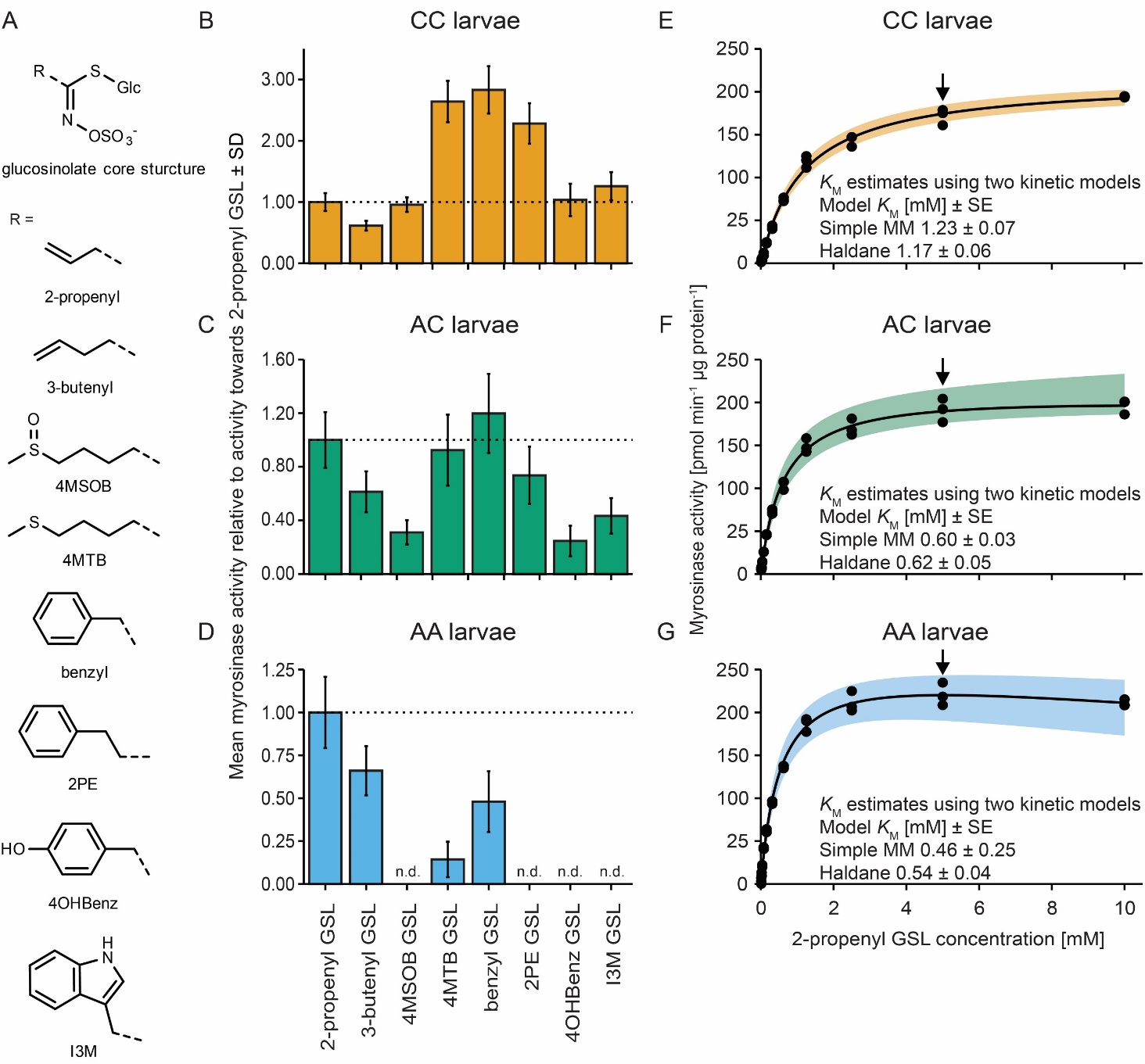


Figure S3

Myrosinase activity in crude protein extracts with different glucosinolate (GSL) substrates (A) differed between *Phyllotreta armoraciae* AA, AC and CC larvae (B-D). Enzyme activity is expressed relative to activity with 2-propenyl GSL, which was set to 1 (indicated with a dotted line). *K*_M_ values toward 2-propenyl GSL in larvae (E-G) were determined based on two different equations. Lines show nonlinear regression used to determine *K*_M_ values based on the Haldane model for single-substrate inhibition (R² > 0.98 for all myrosinases). Coloured bands show 95% confidence intervals. Arrows indicate the highest 2-propenyl GLS concentration used in nonlinear regression to determine the *K*_M_ values based on the Michaelis-Menten model (Simple MM). n.d., not detected; 4MSOB, 4-methylsulfinylbutyl; 4MTB, 4-methylthiobutyl; 2PE, 2‑phenylethyl; 4OHBenz, 4-hydroxybenzyl; I3M, indol-3-ylmethyl.


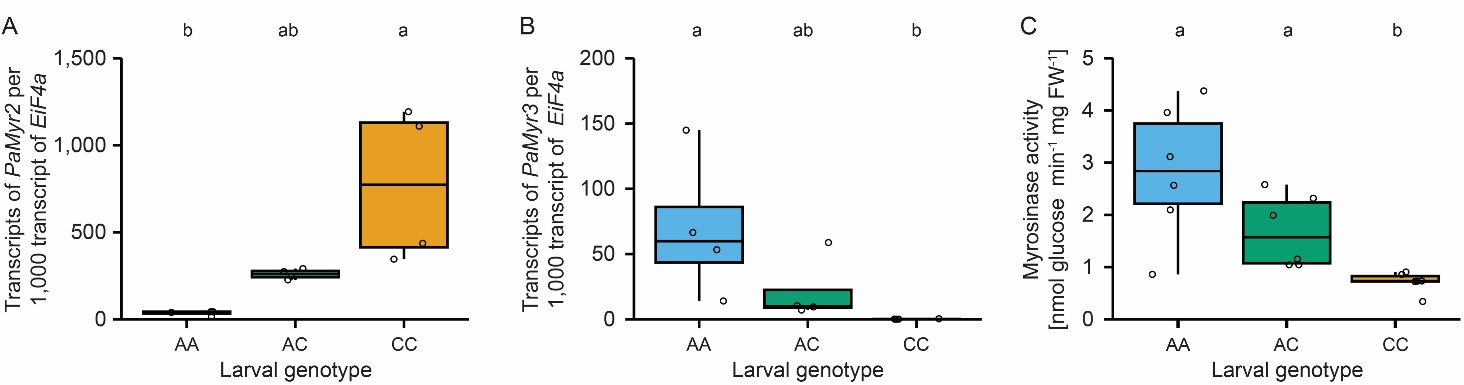


Figure S4

Transcript levels of *PaMyr2* (A) and *PaMyr3* (B) as well as myrosinase activity in crude protein extracts (C) differed significantly among *Phyllotreta armoraciae* AA, AC, and CC larvae sampled as controls for the predation assay with *Harmonia axyridis* (*P* < 0.05; results of statistical analyses are provided in Table S3). Different lowercase letters indicate statistically significant differences between genotypes.


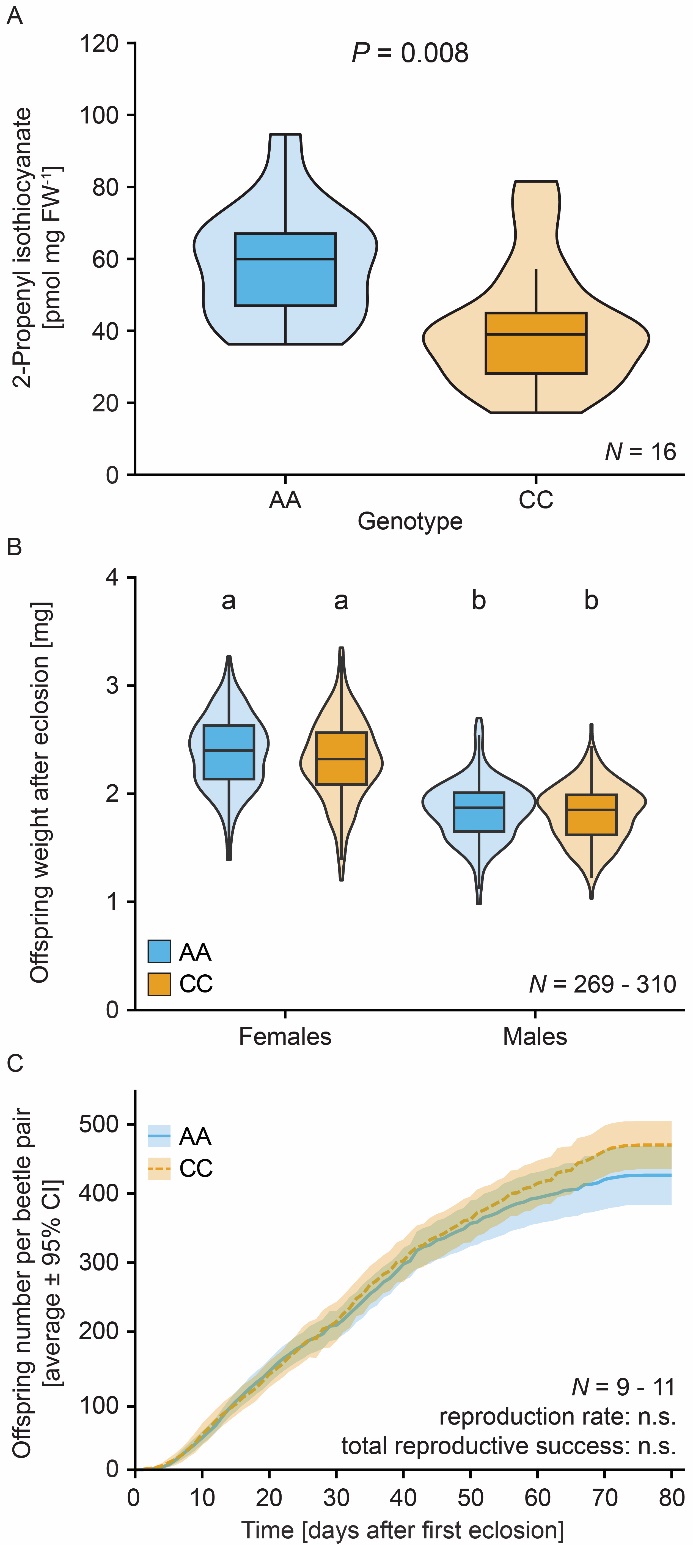


Figure S5

*Phyllotreta armoraciae* AA larvae accumulate significantly higher levels of 2-propenyl glucosinolate-derived hydrolysis products than CC larvae (A). 2-propenyl isothiocyanate accounted for 90% of the total glucosinolate hydrolysis products in extracts. Adult F1 offspring weight (B) and offspring number per mating pair (C) did not differ significantly between AA and CC genotypes. Different lowercase letters indicate statistically significant differences between groups (*P* < 0.05). n.s., not significant

# SI References

Bates, D., Mächler, M., Bolker, B., & Walker, S. (2015). "Fitting Linear Mixed-Effects Models Using lme4". *Journal of Statistical Software*, *67*, no. 1: 1–48. https://doi.org/10.18637/jss.v067.i01

King, R., Buer, B., Davies, T. G. E., Ganko, E., Guest, M., Hassani-Pak, K., Hughes, D., Raming, K., Rawlings, C., Williamson, M., Crossthwaite, A., Nauen, R., & Field, L. (2023). "The complete genome assemblies of 19 insect pests of worldwide importance to agriculture". *Pesticide Biochemistry and Physiology*, *191*: 105339. https://doi.org/10.1016/j.pestbp.2023.105339

Petzoldt, T. (2022). "growthrates: Estimate growth rates from experimental data". *R package version 0.8.4*.
